# Perception of visual advertising in different media: from attention to distraction, persuasion, preference and memory

**DOI:** 10.3389/fpsyg.2014.01208

**Published:** 2014-10-28

**Authors:** Jaana Simola, Jukka Hyönä, Jarmo Kuisma

**Affiliations:** ^1^Cognitive Science, Cognitive Brain Research Unit, University of HelsinkiHelsinki, Finland; ^2^Department of Psychology, University of TurkuTurku, Finland; ^3^Department of Marketing, Aalto University School of BusinessHelsinki, Finland

**Keywords:** advertising, eye movements, attention, memory, ad format, animation, internet, media

Our everyday visual environment is cluttered with advertisements. We come across them in newspapers, magazines, television, and Internet. They can be static, as in print advertisements, or dynamic, as is often the case with TV and Internet ads. The advertising messages are transmitted into the cognitive and affective systems via visual processes. Rather than being a mere input device, the visual processes both voluntarily and involuntarily control the amount and quality of information that is passed onto further mental processing. The effectiveness of advertising therefore critically depends on its ability to attract visual attention. The advertisements should also retain attention long enough in order to allow sufficient encoding of information into the long-term memory.

This Research Topic reviews and further explores processing of visual advertising. We draw together state of the art research on how different ad properties, such as image statistics, surface size of the ads, presence of human faces or animation, and distance of the ads from the editorial content affect ad processing. Furthermore, new measures are proposed to evaluate the capacity of an advertisement to guide observers' gaze and to evaluate the global scanning behavior related to the ads. The Research Topic also presents novel hypotheses and results concerning the impact of narcissism and alcohol intoxication on ad processing. Developmental aspects are taken into account by investigating how children interact with online advertisements. The contributions also successfully cover the diversity of media where visual marketing stimuli appear. The studies review and investigate processing of image and text content of print advertisements as well as advertisements in dynamic media, such as in the Internet or in a digital game environment.

## Conflict of interest statement

The authors declare that the research was conducted in the absence of any commercial or financial relationships that could be construed as a potential conflict of interest.

